# Dual *in vivo* T cell depleted haploidentical hematopoietic stem cell transplantation with post-transplant cyclophosphamide and anti-thymocyte globulin as a third salvage transplant for leukocyte adhesion deficiency with graft failure: a case report

**DOI:** 10.3389/fimmu.2024.1475448

**Published:** 2025-01-08

**Authors:** Azadeh Kiumarsi, Amirarsalan Alaei, Mohsen Nikbakht, Saeed Mohammadi, Mohammad Reza Ostad-Ali, Zahra Rasti, Tahereh Rostami

**Affiliations:** ^1^ Department of Pediatrics, School of Medicine, Children’s Medical Center, Tehran University of Medical Sciences, Tehran, Iran; ^2^ Immunology Research Center, Tabriz University of Medical Sciences, Tabriz, Iran; ^3^ Cell Therapy and Hematopoietic Stem Cell Transplantation Research Center, Research Institute for Oncology, Hematology and Cell Therapy, Shariati Hospital, Tehran University of Medical Sciences, Tehran, Iran; ^4^ Hematologic Malignancies Research Center, Research Institute for Oncology, Hematology and Cell Therapy, Shariati Hospital, Tehran University of Medical Sciences, Tehran, Iran; ^5^ Department of Hematology, School of Allied Medical Sciences, Tehran University of Medical Sciences, Tehran, Iran

**Keywords:** inborn errors of immunity (IEI), leukocyte adhesion deficiency (LAD), hematopoietic stem cell transplantation (HSCT), haploidentical, graft failure (GF)

## Abstract

**Background:**

With recent advances in clinical practice, including the use of reduced-toxicity conditioning regimens and innovative approaches such as ex vivo TCRαβ/CD19 depletion of haploidentical donor stem cells or post-transplant cyclophosphamide (PTCY), hematopoietic stem cell transplantation (HSCT) has emerged as a curative treatment option for a growing population of patients with inborn errors of immunity (IEI). However, despite these promising developments, graft failure (GF) remains a significant concern associated with HSCT in these patients. Although a second HSCT is the only established salvage therapy for patients who experience GF, there are no uniform, standardized strategies for performing these second transplants. Furthermore, even less data is available regarding the outcomes and best practices for a third HSCT as a salvage measure when a second HSCT fails to achieve engraftment.

**Case presentation:**

A 6-year-old boy with leukocyte adhesion deficiency type I (LAD-I) experienced GF after the first and second HSCT from a matched unrelated donor. As a salvage measure, the patient received a dual *in vivo* T-cell depleted haploidentical HSCT. The conditioning regimen for this third HSCT included anti-thymocyte globulin (ATG) and PTCY. Complete donor chimerism was assessed using the short tandem repeat (STR) PCR technique. By day +28 after the transplant, the expression of the leukocyte adhesion molecules CD18, CD11b, and CD11c on the patient’s peripheral blood neutrophils had recovered to over 99%. It remained stable throughout the 18-month follow-up period.

**Conclusion:**

T-cell replete haploidentical HSCT with ATG and PTCY may be a viable salvage option for LAD patients who have rejected prior HSCT.

## Introduction

Leukocyte adhesion deficiency (LAD) syndromes are a group of rare inborn errors of immunity (IEI) (formerly known as primary immunodeficiency) characterized by impaired leukocyte adherence to the endothelium of blood vessels and subsequent migration to extravascular spaces. Individuals with LAD experience tissue neutropenia due to the inability of neutrophils to reach sites of infection (1, 2).

LAD type I (LAD-I) results from biallelic loss of function (LOF) variants in the ITGB2 gene, which encodes a β2-integrin component of the CD11/CD18 molecule (3), leading to CD18 deficiency and inhibiting the leukocyte attachment to endothelial surfaces and proper integrin dimerization. Clinical features of LAD-I include profound neutrophilic leukocytosis, delayed umbilical cord detachment after birth, non-healing ulcers, absence of pus formation at infection sites, and recurrent bacterial and fungal infections, particularly affecting the skin, mucous membranes, and gums (4). The severity of the infectious complications correlates with the level of CD11/CD18 expression in leukocytes. In the absence of allogeneic hematopoietic stem cell transplantation (allo-HSCT), severe LAD-I, which is predominantly classified as <2% neutrophils expressing CD18 as assessed by flow cytometric analysis (5), is characterized by persistent, life-threatening bacterial and other infections, as well as a significant newborn mortality rate. In contrast, the majority of children with mild LAD-I survive into childhood but frequently develop skin and mucosal infections (6).

While allo-HSCT has emerged as a potentially curative approach for patients with severe LAD-I, the condition’s rarity limits the experience and knowledge within individual healthcare facilities (7). Analysis of data from the European Society for Blood and Marrow Transplantation (EBMT) registry revealed that out of 69 LAD-I patients who underwent HSCT between 2007 and 2017, there was an encouraging 3-year survival rate of 84%. Despite these promising outcomes, challenges such as graft failure (GF) and graft-versus-host disease (GvHD) remain significant concerns associated with HSCT in this patient population (8). An HLA-identical sibling is considered the ideal donor for allo-HSCT. However, in the absence of an HLA-matched suitable donor, umbilical cord blood (UCB) or HLA-haploidentical donors have expanded the pool of potential donors for allo-HSCT as attractive alternative options (3).

Here, we present the case of a patient with LAD-I who underwent a successful haploidentical HSCT as a third salvage transplant. This was performed after the patient experienced primary GF following two previous HSCT procedures from HLA-matched unrelated donors.

## Case presentation

A 6-year-old boy diagnosed with leukocyte adhesion deficiency type I (LAD-I) was referred to the center for hematopoietic stem cell transplantation (HSCT). He had a history of recurrent respiratory infections, frequent omphalitis, and otitis. The patient was born to consanguineous parents but had no family history of immunodeficiency. Flow cytometry analysis demonstrated a significant decrease in the expression of CD18 on peripheral blood neutrophils and lymphocytes, with only 0.2% of neutrophils and 2.5% of lymphocytes expressing CD18. Additionally, CD11b and CD11c expression on neutrophils and lymphocytes was also reduced, with levels recorded at 0.6%, 0.7%, 0.1%, and 0.5%, respectively. Given the patient’s clinical course and severe LAD-I diagnosis, HSCT was indicated. Since he did not have a sibling donor, he received a 10/10 HLA-matched unrelated donor allogeneic HSCT with a non-permissive DPB1 mismatch in the graft-versus-host (GVH) direction. Classical HLA alleles of recipients and donors were typed by high-resolution DNA-based methods for class I (A, B, C) and class II (DR, DQ) (**Table 1**). Donor-specific antibodies screening was performed by both Flow crossmatch (FCXM) and solid-phase immunoassay (SPI) to determine mean fluorescence intensity (MFI).

**Table 1:** Human leukocyte antigen (HLA) alleles of recipient and donor.

**Patient**	HLA-A*23:01:01, *31:01:02	HLA-B*49:01:01, *51:01:01	HLA-C*07:01:01, *16:02:01
	HLA-DRB1*10:01:01, *11:04:01	HLA-DQB1*05:01:01, *03:01	HLA-DPB1*14:01:01, *23:01:01
**Unrelated Donor**	HLA-A*23:01:01, *31:01:02	HLA-B*49:01:01, *51:01:01	HLA-C*07:01:01, *16:02:01
	HLA-DRB1*10:01:01, *11:04:01	HLA-DQB1*05:01:01, *03:01	HLA-DPB1*02:01:02, *04:01:01
**Haploidentical Donor (Father)**	HLA-A*68, *31	HLA-B*50, *51	
	HLA-DRB1*10, *03		

**Figure 1:**
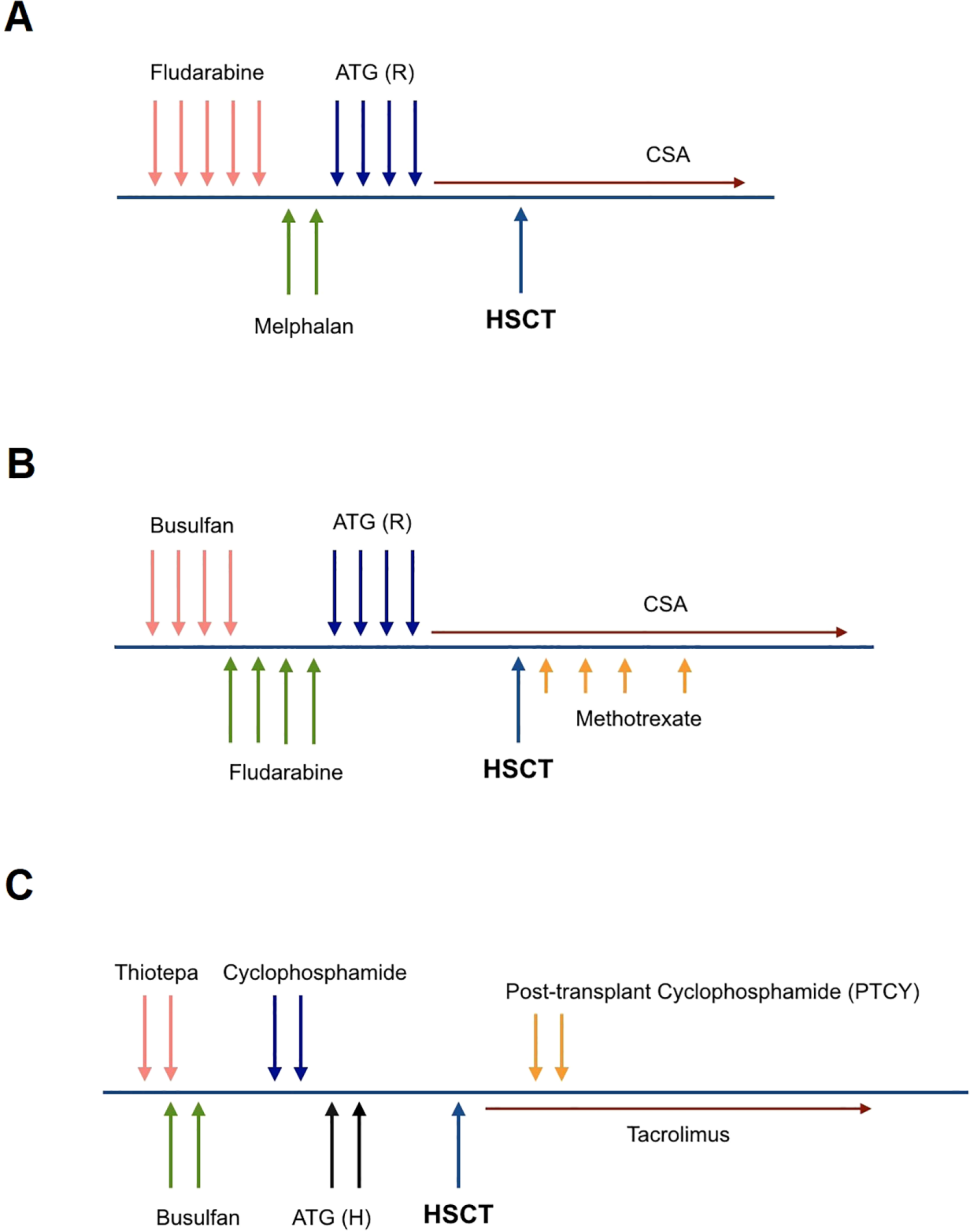
Conditioning regimen for the first transplant (RIC) **(A)**, the second transplant (MAC) **(B)**, and the haploidentical hematopoietic stem cell transplant **(C)**. **(A)** Fludarabine (30 mg/m^2/day × 5 days), Melphalan (70 mg/m^2/day × 2 days), ATG Rabbit; Thymoglobulin (2.5 mg/kg/day from days -4 to -1). **(B)** Busulfan (16 doses; 3.8 mg/kg/day), Fludarabine (30 mg/m^2/day × 5 days), ATG Rabbit; Thymoglobulin (2.5 mg/kg/day from days -4 to -1), Methotrexate (15 mg/m^2^/day on +1 and 10 mg/m^2^/day on +3, +6, +11). **(C)** Thiotepa (5 mg/kg/day × 2 days), Busulfan (8 doses; 3.8 mg/kg/day), Cyclophosphamide (10 mg/kg/day × 2 days), ATG Horse; ATGAM (10 mg/kg/day × 2 days), PTCY (50 mg/kg/day × 2 days).

The graft source was G-CSF mobilized peripheral blood stem cells (PBSC) at a dose of 5 × 10^6/kg CD34+ cells. Pre-transplant donor-specific antibodies (DSA) were negative; the patient and donor were ABO-matched and cytomegalovirus (CMV) seropositive. The preparative regimen was a reduced-intensity conditioning (RIC) regimen consisting of fludarabine (30 mg/m^2/day × 5 days) and melphalan (70 mg/m^2/day × 2 days). Rabbit anti-human thymocyte globulin (ATG-Thymoglobuline, 2.5 mg/kg/day from days -4 to -1) was added to prevent rejection, and the graft-versus-host disease (GvHD) prophylaxis included cyclosporine A (**Figure 1A**).

On day +28 post-HSCT, the patient’s white blood cell (WBC) count was 0.1 × 10^9/L and platelet count was 10 × 10^9/L. Bone marrow examination revealed hypoplasia, and there was no evidence of donor chimerism. Once the diagnosis of primary graft failure was confirmed, cyclosporine A was tapered off.

In similar situations, the preferred approach at the center is to perform a rescue second transplant with a RIC regimen as soon as possible. However, in this case, the patient’s parents refused to accept a second transplant. Therefore, a conservative approach including growth factors was adopted while awaiting hematological recovery. The patient developed autologous hematological reconstitution 40 days after the initial transplant and was discharged from the hospital.

## The second transplant from the same donor

After ten months, the patient was referred again for retransplantation. A second HSCT was planned from the same donor, using a myeloablative conditioning (MAC) regimen and a higher cell dose of 7 × 10^6/kg CD34+ cells. The preparative regimen consisted of busulfan (16 doses; 3.8 mg/kg/day), fludarabine (total dose of 160 mg/kg), and Thymoglobuline, (2.5 mg/kg/day from days -4 to -1). The GvHD prophylaxis included Cyclosporine A and a short course of methotrexate (MTX) (**Figure 1B**). This intensified conditioning regimen and increased cell dose aimed to overcome the previous graft failure and achieve successful engraftment.

Regrettably, the second transplantation was complicated by primary graft failure, leading to a subsequent pulmonary fungal (Aspergillus) infection. The patient’s performance status, as assessed by the Lansky Play-Performance scale, was below 70%, and there were no additional stem cells accessible for transplantation. While salvage HSCT was contemplated and the donor search was promptly initiated, the critical focus was selecting the most suitable donor and devising a safe conditioning regimen.

## Third salvage transplant from haploidentical donor

After recovering from pneumonia and showing significant improvement in performance status, with a Lansky Play-Performance scale score exceeding 80%, the patient underwent a thorough assessment for donor-specific anti-HLA antibodies (DSA). The graft source was T-cell replete G-CSF mobilized peripheral blood hematopoietic stem cells from his haploidentical father with a cell dose of 10 × 10^6/kg CD34+ cells. The conditioning regimen was an immunoablative RIC protocol consisting of thiotepa (5 mg/kg/day × 2 days), busulfan (8 doses; 3.8 mg/kg/day), cyclophosphamide (25 mg/kg/day × 2 days) and horse ATG (ATGAM, 10 mg/kg/day, from days -2 to -1). To prevent GvHD, the patient received post-transplant cyclophosphamide (PTCY; 40 mg/kg/day × 2 days) and cyclosporine A (**Figure 1C**). This haploidentical HSCT with an intensified RIC regimen was the next step in attempting to achieve successful engraftment and reconstitution of the patient’s hematopoietic system after the previous graft failures.

Neutrophil engraftment was achieved on day +13, followed by platelet engraftment on day +19 post-salvage transplant, with complete donor chimerism assessed by short tandem repeat (STR) PCR technique confirmed on day +28. Unfortunately, complications arose in the form of veno-occlusive disease/sinusoidal obstruction syndrome (VOD/SOS) on day +20 of HCT and hemorrhagic cystitis induced by the BK virus. Following successful management of VOD and clearance of BK viremia and viruria, the patient was discharged from the hospital.

## Immune reconstruction after haploidentical HSCT

CD18, CD11b, and CD11c expression on peripheral blood neutrophils recovered to over 99% by day +28 post-transplant and remained stable for 18 months in this patient. This rapid normalization of adhesion molecule expression on neutrophils is a crucial indicator of successful engraftment and immune reconstitution after HSCT.

As part of our center’s post-transplant protocol, B cell, CD8+ T cell, and CD4+ T cell counts are evaluated at the 6-month mark following HSCT to confirm adequate immune reconstitution. Once CD8+ or CD4+ T cell levels reach a minimum of 200 cells/μL and B cell counts are at least 20 cells/μL in peripheral blood, the revaccination process can commence. In this case, the patient reached these minimum cell count thresholds at six months post-HSCT.

However, the patient experienced a complication in the form of steroid-resistant skin chronic graft-versus-host disease (cGvHD) that was successfully managed with the JAK1/2 inhibitor ruxolitinib.

## Discussion

Despite reduced HSCT-related toxicity due to advancements in graft engineering techniques, such as TCRαβ/CD19 depletion or PTCY in HLA-mismatched donors, as well as the use of reduced-toxicity conditioning regimens with treosulfan or busulfan dose adjustment, graft failure remains a serious issue. GF can be mediated by recipient immune cells or loss of donor hematopoiesis due to unknown causes. Notably, the risk of GF is known to be higher in non-malignant disorders compared to malignant conditions. Multicenter studies have reported an incidence of GF ranging from 8 to 16% in distinct IEIs (9–11). Although preventing graft failure is the most advisable approach, there is no clear consensus on the best strategies to reverse this complication. However, some potential approaches to manage graft failure include administering growth factors, waiting for autologous reconstitution (AR), providing an additional hematopoietic progenitor boost, or undergoing a second transplant with a second preparative regimen. Several reports have suggested that a second salvage transplant for graft failure in children can lead to significant transplant-related mortality and seriously compromise overall survival due to prolonged periods of aplasia when the recipient is at a higher risk of infection and hemorrhage (12).

The study conducted by Ayas et al. provides promising insights into using a second hematopoietic stem cell transplant (HSCT) as a salvage therapy for patients with inborn errors of immunity (IEI) who experience graft failure following their initial transplant. The researchers reported that in their cohort of 10 IEI patients undergoing a second HSCT due to primary or secondary graft failure, engraftment was achieved in all cases. Notably, two patients with leukocyte adhesion deficiency (LAD) were among the study participants, and both survived without any evidence of residual disease. Interestingly, the study findings suggest that the timing of the second HSCT may be a crucial determinant of the outcome. Patients who received their second transplant more than six months after the first HSCT demonstrated better survival rates compared to those with a shorter interval between the two procedures (**Supplementary Table S1**) (13).

In a recent study, Laberko et al. reported the outcomes of 47 patients with IEIs who developed early or late graft failure after their initial HSCT and subsequently underwent a salvage transplant. The median time between the first and second HSCT was 5.3 months (range 1.6–45.7), and between graft rejection and the second HSCT, it was 3.3 months (range 0.7–11.3). Six of the 47 patients developed graft rejection after the salvage HSCT, with a median time to graft rejection of 2.4 months (range 2-8). The cumulative incidence of graft failure was 0.15 (95% CI: 0.08–0.29). Interestingly, the incidence of graft failure varied based on the conditioning regimen used for the salvage HSCT: Irradiation-containing reduced-intensity conditioning (RIC): 0.36 (95% CI: 0.17–0.78), Irradiation-containing myeloablative conditioning (MAC): 0.1 (95% CI: 0.02–0.64) and Busulfan/treosulfan-based conditioning: 0.05 (95% CI: 0.06–0.38). However, this difference in incidence based on the conditioning regimen did not reach statistical significance (p = 0.08). Of three patients alive after the second HSCT GF, two received the third HSCTs and were doing well at the last follow-up. This study highlights the challenges associated with graft failure after HSCT for IEI, with a significant proportion of patients experiencing rejection even after a salvage transplant. The data suggest that the choice of conditioning regimen may impact the risk of graft failure, with busulfan/treosulfan-based conditioning potentially associated with lower incidence. However, further research is needed to confirm these findings and optimize salvage HSCT strategies for this high-risk patient population. **Supplementary Table S2** illustrates the outcomes of patients who experienced GF after undergoing a second HSCT, highlighting the subset of patients who subsequently received a third stem cell transplant (9).

The choice of donor for a second HSCT in patients who have experienced GF remains a matter of debate (14). While changing to a different donor may potentially contribute to successful engraftment, there are limited studies investigating the engraftment or survival outcomes of second HSCT based on donor choice, with controversial results. In the setting of graft failure after an HLA-matched HSCT, it is more common to use the same previous donor for a second transplant, as another well-matched donor is rarely available. However, when using the same donor, the engraftment failure rate has been reported to be high. Additionally, the risk of re-collecting stem cells from the initial donor within a short period should be considered. When graft failure occurs after an HLA-mismatched HSCT, the decision to use the same donor or change to a different donor becomes more complex. Changing donors may improve the chances of engraftment, but this approach has not been extensively studied, and the outcomes could be more explicit (15, 16). In summary, while using a different donor for a second HSCT may theoretically enhance engraftment, the limited available data makes it challenging to definitively recommend this approach over using the same donor. The decision should be individualized based on the specific circumstances of each case, including the availability of alternative donors and the risk of re-collection from the initial donor. Further research is needed to provide more guidance on the optimal donor choice for second HSCT in the setting of graft failure.

Our patient experienced primary GF after initial HSCT. We used stem cells from the same donor that had previously been stored for the salvage transplant. Unfortunately, graft failure occurred again after this second transplant. At this point, we decided to use a different donor for the third transplant. However, we could not find a fully matched donor; as a result, the patient underwent a third HSCT using stem cells from a haploidentical donor.

Choosing a safe and effective conditioning regimen is crucial when performing a second or third HSCT after graft failure. The suspected mechanism of GF is an essential factor. An immunoablative conditioning regimen may be preferred for immune-mediated GF, whereas a myeloid-mediated graft failure may be more appropriate. However, there is no consensus on the optimal conditioning regimen for a second HSCT after graft failure. Many transplant centers favor a non-myeloablative RIC regimen for the second HSCT. This maintains sufficient immunosuppression to eradicate residual host cells and promote engraftment. It also helps minimize excessive toxicity, as these patients are very fragile after the first transplant. Myeloablative conditioning may be unnecessary, as the bone marrow is already hypocellular (9, 17–20).

The authors used a MAC regimen for the second HSCT, as the patient had shown autologous recovery after the first graft failure. For the third salvage HSCT, a RIC regimen was considered. This was due to the patient’s very hypocellular marrow and the short time interval since the previous transplant.

With all this, the optimal conditioning regimen for repeat HSCT after graft failure remains an area of uncertainty. Considerations include the mechanism of graft failure and the patient’s clinical status. A RIC approach is often preferred, but the specific regimen should be tailored to the individual patient’s circumstances.

Although there are few reported cases of patients with IEI undergoing a third HSCT as salvage therapy after experiencing graft failure following their first and second HSCT, a recent large retrospective cohort study provides some insights into this high-risk scenario. The study evaluated the outcomes of the second HSCT for IEI in a single center between 2002 and 2022. Of 493 patients who underwent allogeneic HSCT for severe combined immunodeficiency (SCID) or non-SCID IEI, 30 patients (6.0%) required a second HSCT due to graft failure. Notably, three of these patients went on to receive a third HSCT as a salvage measure. The donor source for the third HSCT was the same original donor in two patients and a different donor in one patient. Interestingly, all three patients received T-cell depleted HSCT. One patient underwent a TBI-based conditioning regimen, while no conditioning was used in the patient who received a haploidentical HSCT. Despite the high-risk nature of these cases, the outcomes were encouraging. At the time of reporting, all three patients were alive and had achieved 100% donor chimerism (21). **Supplementary Table S3** presents the key characteristics of these three patients, including details on their underlying diseases, the type of donor used for the third HSCT, the graft source, the conditioning regimen applied, and their survival outcomes.

While the available data is limited, this single-center experience suggests that a third HSCT, particularly with T-cell depletion and individualized conditioning regimens, may be a viable salvage option for select IEI patients who have experienced graft failure after their first and second HSCT.

To the best of our knowledge, this is the first reported case of a patient with LAD who underwent a third HSCT as a salvage treatment. The key aspects of this patient’s management are noteworthy: The patient received a T-cell replete haploidentical HSCT with ATG to mitigate the risk of GF, and PTCY was used for GVHD prophylaxis. By targeting rapidly dividing allo-reactive donor T-cells while preserving regulatory T-cells, PTCY reduces the risk of GVHD and increases the likelihood of engraftment. Moreover, ATG effectively prevents GVHD, particularly chronic GVHD (22–24). The combination of ATG and PTCY has the potential to significantly improve long-term outcomes in HSCT by enhancing the chances of successful engraftment and decreasing the risks of GVHD and non-relapse mortality (NRM) (25, 26).

While ex vivo T-cell-depleted grafts using CD34+ cell enrichment and infusion of mega doses of CD34+ cells from mobilized peripheral blood have shown rapid engraftment in previous studies, this approach has been associated with a higher number of infectious complications due to delayed immune recovery (27).

Our patient achieved successful engraftment and had a favorable outcome despite the high-risk nature of undergoing a third HSCT.

## Conclusion

This case report highlights the potential utility of T-cell replete haploidentical HSCT with ATG and PTCY as a salvage therapy for LAD patients who have experienced graft failure after previous HSCT attempts. However, this report serves as an initial proof-of-concept, and we acknowledge that additional research is crucial to confirm the efficacy and safety of T-cell replete haploidentical HSCT as a salvage therapy and to develop evidence-based guidelines for managing graft failure in IEI patients requiring second or third HSCT.

## Data Availability

The datasets presented in this article are not readily available because Concerns over participant/patient anonymity have prevented the datasets for this article from being publicly available. Requests to access the datasets should be directed to Tahereh Rostami, trostami@sina.tums.ac.ir.
