# Supplementary material for: Dual in vivo T cell depleted haploidentical hematopoietic stem cell transplantation with post-transplant cyclophosphamide and anti-thymocyte globulin as a third salvage transplant for leukocyte adhesion deficiency with graft failure: a case report
Source: Front Immunol. 2025 Jan 8;15:1475448. doi: 10.3389/fimmu.2024.1475448 (PMC11751042; doi:10.3389/fimmu.2024.1475448)
Supplement: Supplementary file 1 [file Table1.docx]

**Table S 1|** Patients with second transplant (13)**.**

| **Disease** | **Type of graft**  **failure** | **Time from 1st to**  **2nd HSCT**  **(months)** | **Donor**  **1st HSCT** | **Donor**  **2nd HSCT** | **Conditioning**  **1st HSCT** | **Conditioning**  **2nd HSCT** | **Engrafted**  **after 2nd**  **HSCT** | **Follow up** |
| --- | --- | --- | --- | --- | --- | --- | --- | --- |
| **LAD** | Primary | 15.8 | MSD | MSD | BU/CY | BU/CY/VP/ATG | Yes | Alive with no evidence of disease |
| **LAD** | Secondary | 8 | UCB | UCB | BU/CY/ATG | CY/TBI/ATG | Yes | Alive with no evidence of disease |
| **Omenn** | Primary | 1.2 | Haploidentical | MRD | CY | BU/CY/VP | Yes | Alive with no evidence of disease |
| **Omenn** | Primary | 2.5 | Mismatched mother | Mismatched mother | BU/CY | ATG | Yes | Alive with no evidence of disease |
| **Omenn** | Primary | 6 | Haploidentical | UCB | BU/CY/ATG | RIC | Yes | Alive with no evidence of disease |
| **BLS** | Secondary | 83 | Matched father | Matched father | BU/CY/VP | RIC | Yes | Alive with no evidence of disease |
| **BLS** | Primary | 24 | MSD | MSD | BU/CY/VP | BU/CY/ATG | Yes | Deceased |
| **BLS** | Primary | 12 | Matched father | Matched mother | BU/CY/VP | RIC | Yes | Deceased |
| **BLS** | Secondary | 12.5 | MSD | MSD | RIC | BU/CY/VP/ATG | Yes | Alive with no evidence of disease |
| **BLS** | Primary | 5 | Mismatched mother | Mismatched mother | RIC | BU/CY/VP/ATG | Yes | Deceased |

ATG, antithymocyte Globulin; BLS, bare lymphocyte syndrome; BU, busulfan; CY, cyclophosphamide; LAD, leukocyte adhesion deficiency; MSD, matched sibling donor; RIC, reduced intensity conditioning; TBI, total body irradiation; UCB, unrelated cord blood; VP, etoposide.

**Table S 2|** Patients who underwent a second HSCT for GF with the subset of patients who subsequently received a third stem cell transplant (9)**.**

| **Diagnosis** | **Age at 2nd**  **HSCT, years** | **Time from 1st**  **to 2nd HSCT, months** | **Conditioning regimen** | **Donor**  **(HLAmatch)** | **Graft type** | **Post-HSCT**  **immunosuppression** | **Time from 2nd HSCT to GF** | **Follow**  **up, time** |
| --- | --- | --- | --- | --- | --- | --- | --- | --- |
| WAS | 2.3 | 3.6 | lymphoid  irradiation/RIC | MUD  (10/10)* | PBSC, PTCY | Tacrolimus | Day +60 | **3rd HSCT,** alive  7.8 years |
| CXCR4 | 1.8 | 5.1 | lymphoid  irradiation/MAC | MUD  (9/10) | PBSC, TCRαβ  depletion | CsA | Day +77 | Death  day +154 |
| CTLA4 | 17.0 | 3.8 | lymphoid  irradiation/MAC | MMRD  (5/10) | PBSC, TCRαβ  depletion | No | Day +61 | Death  day +103 |
| HLH | 1.2 | 1.6 | Flu100,  Thymo10, Cy100 | MMRD  (7/10)* | PBSC, TCRαβ  depletion | No | Day +91 | Alive 2.7 years |
| ELANE | 1.4 | 3.5 | lymphoid  irradiation/MAC | MMRD  (6/10) | BM, PTCY | Tacrolimus | Day +66 | Death, 1.4 years |
| WAS | 1.2 | 3.4 | lymphoid  irradiation/RIC | MUD  (9/10) * | PBSC, PTCY | Tacrolimus | Day +244 | **3rd HSCT**, alive  7 years |
| ELANE | 9.3 | 3.5 | Bu/Treo  MAC | MUD  (10/10) | BM | Tacrolimus/MTX | Day +22 | Death, day +36 |

GF, graft failure; IEI, inborn errors of immunity; MAC, myeloablative conditioning; MMRD, mismatched related donor; MRD, matched related donor; МTX, methotrexate; MUD, matched unrelated donor; PBSC, peripheral blood stem cell; PTCY, post-transplant cyclophosphamide; RIC, Reduced-intensity conditioning.

*the same donor as was used in the first HSCT

**Table S 3|** Patients with GF who received third HSCT (21)**.**

| **Disease** | **HSCT** | **Donor type** | **Graft type** | **Conditioning regimen** | **Follow up** |
| --- | --- | --- | --- | --- | --- |
| **PI3KD** | Second HSCT | MMUD (same as 1st HSCT) | TCRαβ /PBSC | Treo/Flu/TT/ATG | Alive, 4.5 years |
|  | Third HSCT | MMUD (same as 1st HSCT) | TCRαβ /PBSC | Treo/Flu/TT/ATG/TBI |  |
| **PRF-HLH** | Second HSCT | MMUD (same as 1st HSCT) | TCRαβ /PBSC | Treo/Flu/TT/ATG | Alive, 6 months |
|  | Third HSCT | MMUD  (different from the original donor) | TCRαβ/CD19-depleted PBSC graft with add-back  of CD45RO + memory T lymphocyte cells | Treo/Flu/TT/ATG |  |
| **T-B + NK + SCID** | Second HSCT | Haploidentical donor  (same as 1st HSCT) | TCRαβ/ PBSC with add-back of CD45RO + memory T lymphocyte cells | Treo/Flu/TT/ATG | Alive, 7 months |
|  | Third HSCT | Haploidentical donor  (same as 1st HSCT) | TCRαβ/ CD19-depleted | No conditioning |  |

ATG, antithymocyte Globulin; Flu, fludarabine; MMUD, mismatched unrelated donor; PBSC, peripheral blood stem cell; PI3KD, activated PI3K-delta syndrome; PRF-HLH, perforin-deficient hemophagocytic lymphohistiocytosis; SCID, severe combined immune deficiency; TBI, total body irradiation; Treo, treosulfan; TT, thiotepa.
